# Sex-specific difference on anxiety- and depressive-like behavior in neuronal growth regulator 1-knockout mice

**DOI:** 10.1186/s13293-025-00816-2

**Published:** 2026-01-26

**Authors:** So Rok Lee, Eunji Yoon, Sooyeon Baek, Jin Gyeom Kim, Jong-Oh Kim, Su-In Yoon, Soojin Lee, Jin Ah Cho

**Affiliations:** 1 Department of Food and Nutrition, Chungnam National University, 99, Daehak-ro, Yuseong-gu, Daejeon, 34134 South Korea; 2 Research Center for Microbiome-Brain Disorders, Chungnam National University, Daejeon, 34134 South Korea; 3 Department of Sport Science, Hannam University, Daejeon, 34430 South Korea; 4 Department of Microbiology and Molecular Biology, Chungnam National University, Daejeon, 34134 Korea; 5 Glocal life-care Convergence program, Chungnam National University, Daejeon, 34134 South Korea

**Keywords:** Sex differences, Anxiety, Depression, Negr1, Apoptosis, ER stress, Gut–brain axis

## Abstract

**Background:**

Sex differences are evident in anxiety and depression, and women more frequently present with comorbid anxiety and depression alongside gastrointestinal disturbances. This pattern suggests contributions from sex-specific biological mechanisms and gut–brain communication. *Negr1*, a molecule regulating neuronal growth and connectivity, has been linked to depression-relevant behaviors in animal models. However, its mechanisms and potential sex-specific effects remain unclear.

**Methods:**

Behavioral tests were used to assess phenotypes related to depression, anxiety, and learning in male and female wild-type (WT) and *Negr1*^*–/–*^ mice, and molecular assays were performed to evaluate endoplasmic reticulum (ER) stress and apoptosis in the brain, liver, and colon. Behavioral test data were analyzed using a three-way mixed repeated-measures analysis of variance (RM-ANOVA), and molecular data were analyzed using two-way or three-way ANOVA.

**Result:**

*Negr1*^*–/–*^ mice exhibited sex-dependent phenotypes in both central and peripheral systems. Baseline analyses revealed increased intestinal permeability in *Negr1*^*–/–*^ mice and sex-specific patterns of brain-derived neurotrophic factor (*Bdnf*) mRNA expression across multiple time points. Behaviorally, *Negr1*^*–/–*^ mice showed increased anxiety-like behavior, decreased social interaction, and impaired spatial learning in the Morris water maze, regardless of sex. Female *Negr1*^*–/–*^ mice displayed impaired fear learning and increased depression-like behavior, while male *Negr1*^*–/–*^ mice exhibited heightened anxiety-like responses. At the molecular level, ER stress marker spliced X-box binding protein 1 (*Xbp1s)* mRNA was upregulated in peripheral tissues of males but downregulated in females. Apoptosis analysis revealed enhanced caspase-3 activation in peripheral tissues of female *Negr1*^*–/–*^ mice, while males showed no significant changes. Brain tissue showed no significant apoptotic alterations in either sex.

**Conclusions:**

This study demonstrates that, in *Negr1*^*–/–*^ mice, both sexes show general behavioral alterations. However, female *Negr1*^*–/–*^ mice exhibit greater vulnerability to fear learning impairments and depression-like behavior, whereas males *Negr1*^*–/–*^ mice show heightened anxiety responses. These behavioral differences were associated with opposing ER stress responses and differential apoptotic signaling between the sexes in peripheral tissues. Our findings highlight the importance of considering sex as a biological variable in depression research and suggest that *Negr1* plays a crucial role in the sex-specific pathophysiology of psychiatric disorders through complex mechanisms spanning central and peripheral systems.

**Supplementary Information:**

The online version contains supplementary material available at 10.1186/s13293-025-00816-2.

## Plain Language summary

This study investigated how loss of the neuronal growth regulator 1 (*Negr1*) affects depression-related behaviors differently by sex in the *Negr1* knockout mouse model. Both male and female mice in the absence of *Negr1* exhibited reduced social interaction and impaired long-term learning abilities. However, the specific types of behavioral problems differed markedly by sex. Female *Negr1*^*–/–*^ mice exhibited signs of depression, including increased immobility when suspended by their tails and difficulty learning to avoid fearful situations. In contrast, male *Negr1*^*–/–*^ mice showed primarily anxiety-like behaviors, such as avoiding open spaces.

At the molecular level, we found opposite patterns between sexes in how their bodies respond to cellular stress. In peripheral organs like the liver and intestine, males activated protective stress-response pathways, while females showed reduced protective responses and increased cell death. Interestingly, these sex-specific differences were seen only in peripheral tissues, not in the brain itself.

These findings suggest that the gut–brain connection works differently in males and females when the Negr1 gene is absent. Males appear to mount protective responses in peripheral tissues while experiencing anxiety, whereas females show increased vulnerability to cellular damage in peripheral tissues alongside depression-like behaviors. This research highlights why understanding sex differences is crucial for developing more effective, personalized treatments for depression and anxiety disorders.

**Supplementary Information:**

The online version contains supplementary material available at 10.1186/s13293-025-00816-2.

## Highlights

- *Negr1*^–/–^ mice showed increased intestinal permeability, and sex-specific *Bdnf* mRNA expression across multiple time points.
- Female *Negr1*^–/–^ mice showed impaired fear learning and increased depression-like behavior, while males *Negr1*^–/–^ mice displayed stronger anxiety-like behavior.
- *Xbp1s* mRNA expression increased in peripheral tissues (liver and colon) of male *Negr1*^–/–^ mice but decreased in females, with no changes in phosphorylated eukaryotic initiation factor 2α (p-eIF2α) or C/EBP homologous protein (*Chop*) expression.
- Female *Negr1*^–/–^ mice exhibited enhanced caspase-3 cleavage in peripheral tissues (colon), while males showed no significant changes: brain tissue showed no apoptotic alterations in either sex.

**Supplementary Information:**

The online version contains supplementary material available at 10.1186/s13293-025-00816-2.

## Background

Anxiety and depressive disorders typically have peak onset in adolescence and early adulthood [1], with women at a substantially higher risk than men [2]. These differences suggest that sex-specific biological mechanisms underlie depression pathophysiology [3], yet the molecular mechanisms remain poorly understood.

NEGR1 is a glycosylphosphatidylinositol-anchored adhesion protein of the immunoglobulin superfamily LAMP/OBCAM/Neurotrimin (IgLON) family that facilitates neuronal cell recognition and neurite outgrowth [4–6]. NEGR1 is expressed in brain regions critical for mood regulation, including the hippocampus, prefrontal cortex, and hypothalamus, where it maintains structural and functional integrity [7–10]. Importantly, beyond the brain, NEGR1 is also expressed in various peripheral tissues such as the liver and intestine, positioning it as a potential mediator of gut–brain communication [11]. Genome-wide association studies (GWAS) have identified *NEGR1* as a genetic risk factor for depression [12]. In mouse models, *Negr1* deficiency elicits depression-like behaviors [13], and other studies have reported social withdrawal and cognitive impairments [9]. However, previous studies have not systematically examined sex differences in *Negr1*-related phenotypes, limiting our understanding of how this gene contributes to sex-specific depression mechanisms.

Depression is increasingly recognized as a systemic condition with prominent gastrointestinal (GI) disturbances [14]. Patients with depression frequently present with nausea, constipation, and other GI disturbances, with over 80% of elderly depressed patients reporting such symptoms [15, 16]. Women also tend to exhibit more pronounced symptom profiles, including GI disturbances. Studies have reported that, in women, the presence of gastrointestinal disorders is associated with increased odds of anxiety and depression [17]. This suggests that sex may shape clinical presentation through the gut–brain axis. These findings implicate sex-dependent gut–brain mechanisms, potentially mediated by hormonal effects on the microbiota, barrier integrity, and immune signaling [18–21].

Additionally, cellular stress responses including ER stress and apoptosis show sex-specific regulation [22] and are elevated in Major depression disorder (MDD) patients, particularly in brain regions and peripheral tissues [23–25]. BDNF, a key neuroplasticity regulator, is significantly downregulated in depression, contributing to synaptic dysfunction [26, 27].

Given that the lack of *Negr1* produces depression-like behaviors and its expression spans both central and peripheral tissues, we hypothesized that *Negr1* knockout would reveal sex-specific vulnerabilities through differential effects on behavior, neuroplasticity markers, and peripheral stress responses. To test this hypothesis, we conducted comprehensive behavioral, molecular, and physiological analyses in male and female *Negr1*^*–/–*^ mice, with a particular focus on anxiety- and depression-related behaviors and their underlying mechanisms involving the gut–brain axis.

## Materials and methods

### Animals and experimental design

Three separate cohorts were used in this study. Cohort 1 included the wild-type (WT; 8 weeks: 8 males, 6 females; 11 weeks: 8 males, 5 females; 14 weeks: 13 males, 16 females) and *Negr1*^*–/–*^ (8 weeks: 4 males, 4 females; 11 weeks: 4 males, 4 females; 14 weeks: 11 males, 10 females) [13] mice sacrificed at 8, 11, and 14 weeks for analyses of baseline corticosterone (CORT) levels. Cohort 2 consisted of WT (8 weeks: 4 males, 3 females; 18weeks: 4 males, 4 females) and *Negr1*^*–/–*^ (8 week: 5 males, 4 females; 18 weeks: 6 males, 5 females) mice sacrificed at 8 and 18 weeks for analyses of baseline *Bdnf* expression. Behavior tests were not performed on Cohort 1 and 2. Cohort 3 consisted of WT (6 males, 10 females) and *Negr1*^*–/–*^ (8 males, 10 females) mice that underwent the complete behavioral tests from 8 to 30 weeks of age, followed by sacrifice at 32 weeks for molecular analyses.

All housing and testing conditions were identical between cohorts. The mice were housed in standard-sized laboratory cages, with 2–5 animals per cage in our study. The light/dark cycle was maintained at 12:12 h (lights on at 7:00, lights off at 19:00), and they had ad libitum access to water and food pellets in animal colonies maintained at a temperature of 22 ± 1 °C. All procedures were approved by the Chungnam National University Animal Experiment Ethics Committee (202206 A-CNU-116).

### Behavioral tests

**Fig. 1:**
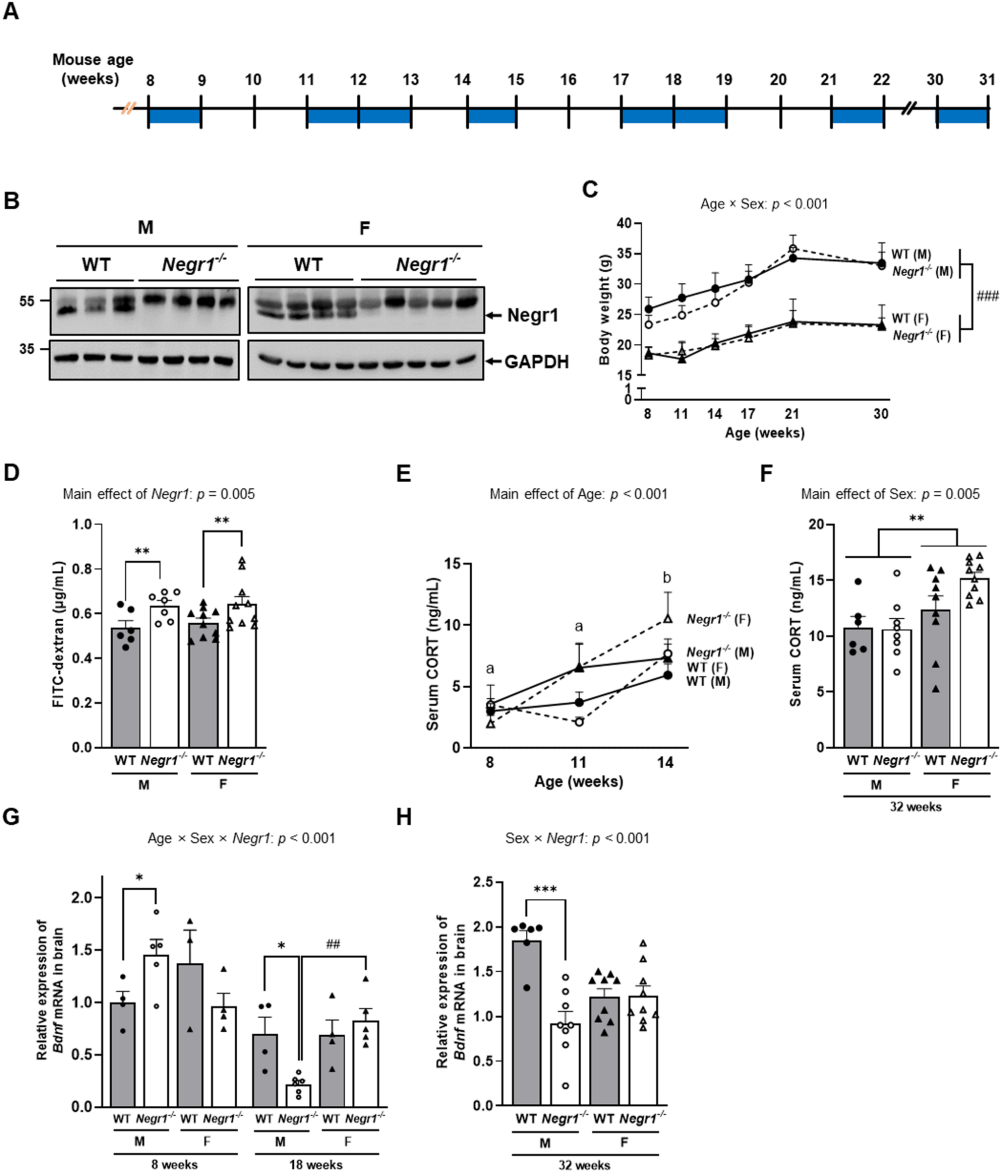
*Negr1*^–/–^ mice show sex-dependent characteristics. **A.** This schedule provides a schematic representation of the experimental design and indicates the behavioral test period (blue box). **B.** Negr1 protein expression in brain tissue, confirmed by Western blot. **C.** Body weight was measured over time, with values recorded on the day before each behavioral testing. **D**. Intestinal permeability assessed using plasma FITC–dextran levels. **(E)** Serum CORT levels were measured in Cohort 1 (at 8, 11, and 14 weeks of age). **(F)** Serum CORT levels were measured in Cohort 3 (at 32 weeks, endpoint). **(G)** *Bdnf* mRNA expression in the brain was measured in Cohort 2 (at 8 and 18 weeks of age). **(H)** *Bdnf* mRNA expression in the brain was measured in Cohort 3 (at 32 weeks, endpoint). RM-ANOVA was used for **C**, two-way ANOVA for **D**,** F** and **H**, and three-way ANOVA (age x sex x genotype) for **E** and **G**. Post hoc comparisons were performed using Sidak’s test. Male vs. female comparisons: ^##^ *p <* 0.01 and ^###^ *p <* 0.001. WT vs. *Negr1*^–/–^ comparisons: * *p <* 0.05, ** *p <* 0.01 and *** *p <* 0.01. Letters (a–c) denote the significant main effect of age. Data are presented as mean ± SEM. M, males; F, females

All behavioral experiments commenced at 8 weeks of age, with the detailed time schedule presented in Fig. 1A. Body weight was measured the day before behavioral experiments, and mice were adapted to the experimental room and lighting conditions for 1 h before the experiment. The tests were conducted in the following order: open field test (OFT), elevated plus maze (EPM), tail suspension test (TST), three-chamber social interaction test (3-SIT), Y-maze, passive avoidance test (PAT), sucrose preference test (SPT), and Morris water maze (MWM). MWM was conducted at weeks 12 and 18. Mice were allowed a 24-hour rest period between the behavioral tests.

### OFT

We assessed anxiety levels using the OFT. OFT apparatus consisted of a gray acrylic square arena (diameter = 40 cm), divided into a central area (diameter = 20 cm) and peripheral zones. Distance and time spent in the central and peripheral zones were recorded for 5 min using a video camera positioned above the OFT apparatus and analyzed using the SMART 3.0 video tracking system (Panlab Harvard Apparatus, Holliston, MA, USA). The formula used was: D_center_ (%) = D_center_ / (D_center_ + D_peripheral_) x 100. This formula calculates the percentage of distance traveled in the center zone. It also applies to calculating the percentage of time spent in the center zone, as well as the percentage of distance and time spent in the peripheral zone.

### EPM

The EPM measures the level of anxiety-like activity of the rodents. The apparatus consisted of a cross-shaped maze with two open arms arranged opposite each other and two closed arms with side walls extending 15 cm in height. Distance and time spent in the open arm zones and the closed arm zones were recorded for 5 min using a video camera positioned above the maze and analyzed using the SMART 3.0 video tracking system (Panlab Harvard Apparatus). Formula: D_open_ (%) = D_open_ / (D_open_ + D_closed_) x 100 was employed. This formula calculates the proportion of distance traveled in the Open arm. It is also applied likewise to calculate the ratio of time spent in the open arm, as well as the ratio of distance and time spent in the closed arm. In this test, distance and time spent in the open arm zone were considered as indicators reflecting the level of anxiety-like activity.

### TST

The TST is the representative test used in rodent studies to evaluate depression-like behavior, with immobility considered a marker of behavioral despair. Mice were suspended approximately 20 cm above the floor using adhesive tape placed ~ 1 cm from the tip of the tail. Each trial lasted for 6 min, which included a 2 min habituation period followed by a 4 min phase during which immobility was assessed. All sessions were continuously video-recorded to allow subsequent analysis. Immobility was defined as the absence of movement, apart from minor motions required for respiration, while the animal remained hanging passively. Furthermore, to minimize potential visual interference, mice were tested individually in isolation. Finally, the total duration of immobility was measured in seconds.

### SPT

The SPT is a behavioral assay used in rodent models to measure anhedonia, or the reduced ability to experience pleasure, which is a symptom of depression. Mice were given free access to both a 1% (w/v) sucrose solution and plain water for 48 h of acclimatization. After this habituation phase, they were deprived of water for 24 h before undergoing the SPT. During the test, two identical bottles—one with 1% sucrose solution and the other with water—were made available for 24 h, with their positions switched after 12 h to avoid side bias. Fluid intake (g) from each bottle was measured, and sucrose preference (%) was calculated as [sucrose intake ÷ (sucrose intake + water intake)] × 100.

### 3-SIT

The 3-SIT was performed to evaluate social interaction and social novelty preference in rodents [28]. The test consisted of two consecutive phases. In Phase 1: Social Exploration, an experimental mouse was first placed in the central chamber for a 5 min acclimation period. A novel mouse (Stranger 1) was then introduced into a wire-mesh enclosure in one of the side chambers, while the opposite side chamber was left empty. The subject mouse was returned to the central chamber and allowed to explore all three chambers for 10 min. In Phase 2: Social Novelty Preference, a second novel mouse (Stranger 2) was introduced into the previously empty side chamber. The subject mouse was again allowed to explore for 10 min. The cumulative time spent in each chamber, as well as the cumulative time spent interacting with an empty wired cup (object) or the wired cup containing the social-partner mouse was measured. Interaction with either was defined as when the experimental mouse was in close proximity (~ 1 cm) and nose-oriented towards the cups. In the formula: social index = T_s_ / (T_s_ + T_e_), T_s_ is the time spent in the detection zone surrounding the stranger mouse and T_e_ is the time the subject mouse detected around the empty cage. For the social novelty test, the formula: novel index = T_n_ / (T_n_ + T_f_) was used to score the novel index. T_n_ represents the time to interact with the novel mouse and T_f_ means the time in the detection zone of the cage with the familiar mouse. All behavioral data were automatically quantified using the SMART 3.0 video tracking system (Panlab Harvard Apparatus).

### Y-maze

The Y-maze test was performed to test short-term spatial memory of the mouse. The test was a single trial for each animal and 10 min in duration. The animal was placed at the end of one arm facing the center. An overhead camera was set to record the number of entries into the arms and alterations, the sequence of arm entries. An alternation was defined as a consecutive entry into three different arms (e.g., ABC, BAC, CAB etc.). The alternation percentage was calculated using the following formula: Alternation percentage = [Number of alternations / (Total number of arm entries − 2)] × 100. The data collection is automatically assessed by a SMART 3.0 video tracking system (Panlab Harvard Apparatus).

### PAT

The PAT was conducted to assess fear-learning memory using a shuttle box divided into bright and dark compartments [29], connected and separated by a door that could be opened and closed. On the first day, the animals had an adaptation period of 1 min in the light space. After that, the door was opened to allow movement into the dark space, and the moment the animal entered the dark space, an electric shock of 0.8 mA was applied to the foot for 5 s. After 1 min, the animal was taken out of the box and returned to its original cage. After 24 h, the same procedure was repeated to assess the learned avoidance response, but this time without electric shock, and the time taken for the animal to enter the dark space was measured. If the animal did not enter the dark space within 5 min, it was I ended the experiment without waiting any longer.

### MWM

Spatial learning and long-term memory in mice were evaluated using the MWM at 12 and 18 weeks of age. The maze consisted of a circular pool (136 cm in diameter, 60 cm high) was filled with opaque water supplemented with skim milk to prevent visibility to a depth of 20 cm, and the water temperature was maintained at 23–25 °C throughout the testing period. The circular escape platform with 10 cm in diameter, was positioned at the center of one quadrant of the pool, submerged 1.5 cm below the water surface. The experiment consisted of a 5-day training phase and a single probe trial on the sixth day. During the training phase, mice were trained twice daily with a 20 min interval between sessions. In each training session, mice were given 60 s to find the hidden platform, followed by a 30 s rest period on the platform. The time taken to locate the platform was recorded after each training session. On the sixth day, a probe trial was conducted, during which mice were allowed 60 s to search for the platform in the quadrant where it had been located during training. The time spent in the target quadrant was recorded as a measure of spatial memory retention.

### Histological analysis

After measuring the length of the collected colon, the colon tissues were fixed in 10% formalin for histological analysis. Fixed samples were embedded in paraffin, sectioned, and stained with hematoxylin and eosin (H&E) and Alcian blue (AB) by T&P Bio (Gyeonggi-do, Korea). Images of stained tissues were obtained with an optical microscope (Olympus, Tokyo, Japan).

### Intestinal permeability assay

Intestinal permeability was evaluated using 4 kDa Fluorescein isothiocyanate–dextran (FITC-dextran, Sigma-Aldrich, St. Louis, MO, USA). The mice were fasted for 4 h and then FITC-dextran (600 mg/kg body weight) was oral-gavaged. Four hours later, whole blood was collected by orbital blood collection and the fluorescence intensity of FITC-dextran in plasma was analyzed using a multimode plate reader (BMG Labtech, Ortenberg, Germany).

### Measurement of serum CORT level

Serum CORT was measured using a commercial Enzyme-Linked Immunosorbent Assay (ELISA) kit (Novus Biologicals, Littleton, CO, USA) according to the manufacturer’s protocol. For baseline measurements in Cohort 1, serum samples were collected at 8, 11, and 14 weeks of age in the animal housing room to minimize the effects of acute stress (handling and transport). Cages were gently removed from the rack and placed immediately on the workbench. The time from initial cage handling to completion of blood collection was limited to approximately 5 min. All blood collections were performed by the same experimenter familiar with the animals. To account for the circadian rhythm of CORT, all samples were collected at a consistent time during the photoperiod (e.g., 1–2 PM). For the 32-week cohort (Cohort 3), after completing the final behavioral test, animals were allowed to rest in their cages for 48 h without further testing. Blood for CORT determination was then collected via cardiac puncture after anesthesia. Samples were diluted, pretreated, and centrifuged. Standards were prepared by serial dilution. After incubation with the primary antibody, wells were washed, reacted with substrate, and the reaction was stopped. Absorbance was read at 450 nm with a 570 nm reference using a microplate reader. Serum CORT concentrations were calculated from a four-parameter logistic (4-PL) standard curve.

### RNA extraction, cDNA synthesis and polymerase chain reaction (PCR)

The tissues were homogenized with TRI reagent (MRC, Cincinnati, OH, USA) using the GentleMACS™ Dissociator (Miltenyi Biotec, Bergisch Gladbach, Germany), the total RNA concentration was assessed using a Nanodrop spectrophotometer (Thermo Fisher Scientific Inc., Waltham, MA, USA), and cDNA synthesis was carried out using an RT-kit (BioFACT, Daejeon, Korea). Real-Time quantitative polymerase chain reaction (RT-qPCR) was performed using 2X Real-Time PCR Master Mix containing SYBR Green (Enzynomics, Daejeon, Korea) on real-time PCR system (Agilent, Santa Clara, CA, USA). For reverse transcription polymerase chain reaction (RT-PCR), cDNA was mixed with 2X Taq Basic PCR Master Mix 2 (BioFACT), subjected to PCR amplification, and then loaded onto either 2% agarose gel or a 3% agarose gel. Gel images were captured under UV light using gel documentation system (ATTO, Tokyo, Japan) and quantified using Image J software (NIH Image, Bethesda, MD, USA). Primer sequences for RT-qPCR and RT-PCR are listed in Supplementary Table. S1 and Supplementary Table. S2.

### Western blotting

Tissues were homogenized in RIPA buffer (Thermo Fisher Scientific) using a GentleMACS™ Dissociator (Miltenyi Biotec Co.), followed by measurement of protein concentrations using a bicinchoninic acid (BCA) protein assay (Thermo Fisher Scientific). Subsequently, proteins were separated on polyacrylamide gels and transferred onto nitrocellulose membranes (Cytiva, Marlborough, MA, USA). The membranes were blocked with 5% skim milk in twin tris buffered saline (TTBS). The primary antibodies used included Negr1 (Santa Cruz Biotechnology, Santa Cruz, CA, USA, 1:1000), p-eIF2α (Cell signaling Technology, Danvers, MA, USA, 1:1000), eIF2α (Cell signaling Technology, 1:1000), caspase-3 (Cell signaling Technology, 1:1000), vinculin (Santa Cruz Biotechnology, 1:5000) and Glyceraldehyde-3-phosphate dehydrogenase (GAPDH, Thermo Fisher Scientific, 1:1000). Following primary antibody incubation overnight, the membranes were washed three times with 1× TTBS and then incubated with secondary goat anti-rabbit antibody or anti-mouse antibody (Thermo Fisher Scientific) for 1 h. Visualization was carried out using chemiluminescence western blot reagents (Thermo Fisher Scientific) and the ChemiDoc system (ATTO, Tokyo, Japan). Western blot bands were quantified using ImageJ software (National Institutes of Health, Bethesda, MD, USA).

### Statistical analysis

Statistical analyses were performed using SPSS 29.0 software (IBM Corp., Armonk, NY, USA) and graphs were generated in GraphPad Prism 9.0 software (GraphPad, Boston, MA, USA). Data are presented as mean ± standard error of the mean (SEM). For single time-point measurements, two-way ANOVA was used with sex and genotype as factors.

Three-way ANOVA was performed with age, sex, and genotype as factors for serum CORT level (Cohort 1) and *Bdnf* expression (Cohort 2) since the samples were harvested at the time of sacrifice and these groups were not under repeated behavior tests during the experimental period. When significant interactions were detected in all ANOVAs, Sidak’s post hoc tests for multiple comparisons were performed. Statistical significance was set at *p* < 0.05. All statistical values (*F* statistics, degrees of freedom, and *p*-values) are reported in the results section.

For behavioral experiments conducted at multiple time points using the same animals (Cohort 3), mixed RM-ANOVA was performed with age as the within-subjects factor and sex and genotype as between-subject factors. We employed a structured presentation strategy: main figures focus on phenotypes with significant sex × genotype interactions, while Supplementary figures provide full longitudinal data and results without significant group effects for completeness. Mixed 3-way RM-ANOVA analyzes data from the same subjects across multiple time points and, in a single model, tests the effects of time, group factors (genotype and sex), and their interactions. When overall effects were significant, we used Sidak’s post hoc tests to identify which pairwise groups differed while minimizing the risk of false positives (Type I error) from multiple comparisons. Because our study included repeated measurements and potential sex × genotype interactions, we applied RM-ANOVA to evaluate overall patterns and Sidak’s post hoc tests for targeted pairwise comparisons.

## Result

### General characteristics of *Negr1*^*–/–*^ mice

To investigate the general characteristics of *Negr1*^*–/–*^ mice used in our study, we validated Negr1 protein expression by showing no 45 kDa Negr1 protein in the *Negr1*^*–/–*^ mice in the brain tissues (Fig. 1B).

Body weight analysis using RM-ANOVA revealed no significant three-way interaction among age, sex, and genotype (*F*_2.56, 71.66_ = 0.467, *p* = 0.510). However, a significant age x sex interaction was observed (*F*_2.56, 71.66_ = 20.704, *p* < 0.001), indicating that both males and females gained weight over time, with males showing greater weight gain than females throughout the study period (Fig. 1C).

Intestinal permeability assessment showed a significant main effect of genotype (*F*_1,29_ = 9.478, *p* = 0.005), with *Negr1*^–/–^ mice exhibiting significantly higher FITC-dextran levels than WT mice, suggesting relatively leaky gut in *Negr1*^–/–^ mice regardless of sex (Fig. 1D).

Due to the fundamental difference in experimental conditions, specifically, Cohort1 underwent no behavioral testing (baseline) while Cohort 3 was subjected to repeated behavioral assessments (chronic stress), serum CORT concentrations were analyzed separately for the two cohorts. First, in Cohort 1, which did not undergo behavioral testing, basal CORT levels were analyzed using a three-way ANOVA with age, sex, and genotype as factors. This analysis revealed a significant main effect of age on CORT levels (*F*_2, 81_ = 12.091, *p* < 0.001), with no significant main effects of sex or genotype and no significant interactions (Fig. 1E). In Cohort 3, CORT was measured only at 32 weeks after completion of repeated behavioral testing. Although 48 h rest period was given to mitigate acute stress, these levels likely reflect the cumulative influence of chronic experimental stress. Two-way ANOVA (sex X genotype) revealed only a significant main effect of sex, with CORT concentrations being lower in males than in females (*F*_1, 29_ = 10.023, *p* = 0.004), and no significant sex × genotype interaction (Fig. 1F).

Similarly, *Bdnf* mRNA expression was analyzed separately in Cohorts 2 and 3 because the experimental conditions differed between cohorts. In Cohort 2, which did not undergo behavioral testing, *Bdnf* mRNA expression was assessed at baseline and analyzed using a three-way ANOVA (age x sex x genotype). This analysis revealed a significant age × sex × genotype interaction (*F*_1, 27_ = 15.634, *p* < 0.001). Post hoc tests at specific time points elucidated the pattern (Fig. 1G). At 8 weeks, a significant sex difference was observed within the *Negr1*^–/–^ group, with males showing higher *Bdnf* expression than females (*p <* 0.05). At 18 weeks, the pattern reversed; male WT mice exhibited significantly higher *Bdnf* expression compared to male *Negr1*^–/–^ mice (*p <* 0.05), resulting in significantly lower expression in male *Negr1*^–/–^ compared to female *Negr1*^–/–^ (*p <* 0.01). In Cohort 3, *Bdnf* mRNA was measured at 32 weeks after completion of repeated behavioral testing, and a two-way ANOVA showed a significant sex × genotype interaction (*F*_1, 28_ = 17.362, *p* < 0.001) (Fig. 1H). Post hoc comparisons indicated that at 32 weeks, male WT mice exhibited significantly higher *Bdnf* expression than male *Negr1*^–/–^ mice (*p* < 0.05) and also higher expression than female WT mice (*p* < 0.05), whereas no significant genotype differences were observed in females. There was no significant difference in the colon length by genotype nor sex (Supplementary Fig.S1).

### General behavioral phenotypes of *Negr1*^–/–^ mice

**Fig. 2:**
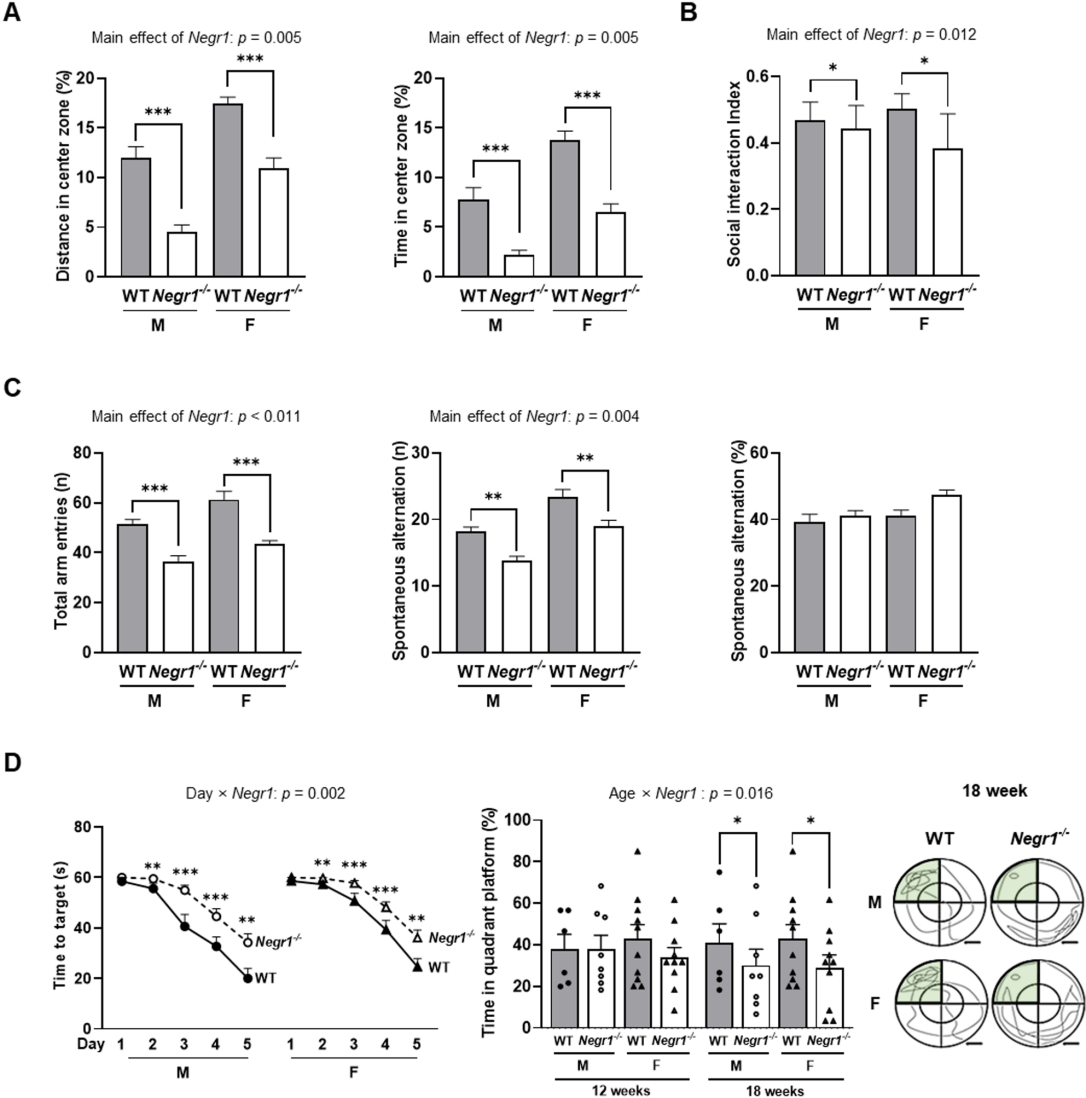
Both sexes of *Negr1*^–/–^ mice exhibited anxiety, reduced sociability, and impaired spatial learning. **(A)** Assessment of center zone distance (%) and time (%) relative to total distance and time, measured in the open field test (OFT). **(B)** Assessment of social interaction ratio in the 3-chnaber social interaction test (3-SIT). **(C)** Assessment of total number of arm entries (left), the number of spontaneous alternations (middle), and the percentage of spontaneous alternations (right) in the Y-maze. **(D)** Latency to the hidden platform during the five-day Morris water maze (MWM) training phase (left), percentage of time in the target quadrant on day 6 (middle), and representative swim paths from the 18-week probe trial (right). Data in this figure were obtained from the Cohort 3 and summarize genotype effects across all tested ages (8–30 weeks). These assays showed a significant main effect of genotype but no significant sex x genotype interaction. The data are presented separated by sex to maintain consistency with the study’s focus on sex-specific differences. RM-ANOVA was used to assess group main effects and interactions, with Sidak’s post hoc comparisons. WT vs. *Negr1*^–/–^ comparisons: * *p <* 0.05, ** *p <* 0.01, and *** *p <* 0.001. Data are presented as mean ± SEM. M, males; F, females

To characterize the behavioral phenotypes associated with *Negr1*, we conducted repeatedly comprehensive behavioral assessments from 8 to 30 weeks of age. The results of behavioral tests showing significant genotype main effects but no sex × genotype interaction is presented in Fig. 2, while complete temporal data and negative findings are included in Supplementary Fig. S2. Similarly, the longitudinal data for all indices of PAT, TST, and EPM are provided Supplementary Fig. S3 for comprehensive analysis across all tested ages (weeks 8 to 30).

In the OFT, RM-ANOVA revealed significant main effects of genotype for both center distance (*F*_1,30_ = 62.278, *p <* 0.001) and center time (*F*_1,30_ = 41.696, *p <* 0.001), with *Negr1*^–/–^ mice spending significantly less distance and time in the center zone compared to WT mice across all time points (Fig. 2A) No significant sex × genotype interactions were detected (distance: *F*_1,30_ = 0.003, *p =* 0.956; time: *F*_1,30_ = 0.685, *p =* 0.414), indicating this anxiety-related behavior was common phenotype in both male and female *Negr1*^–/–^ mice. Total distance traveled showed no significant genotype differences (*F*_1,30_ = 0.685, *p =* 0.414), confirming that reduced center exploration was not due to locomotor impairments (Supplementary Fig. S2A).

In the 3-SIT, a representative behavior test for social interaction, *Negr1*^–/–^ mice showed a significantly lower social interaction index compared to WT mice (main effect of genotype: *F*_1,27_ = 7.249, *p =* 0.012), with no significant sex × genotype interaction (*F*_1, 27_ = 1.827, *p =* 0.188) (Fig. 2B). However, novel index, which represents the preference for a new social partner over a familiar one, did not show a main effect of genotype (*F*_1, 27_ = 1.009, *p =* 0.323) nor significant difference between groups (sex × genotype: *F*_1, 27_ = 3.088, *p =* 0.089) (Supplementary Fig. S2B).

Y-maze analysis revealed significant main effects of genotype for both total arm entries (*F*_1, 30_ = 19.703, *p <* 0.001) and spontaneous alternation (*F*_1, 30_ = 9.974, *p =* 0.004), with no significant sex × genotype interactions (Total arm entries: F_1, 30_ = 0.142, *p* = 0.709; alternation: *F*_1, 30_ = 0.000, *p =* 0.986). Also, for the percentage of spontaneous alternations, neither the main effect of genotype nor the sex × genotype interaction was significant (*F*_1, 30_ = 0.521, *p* = 0.476), indicating that short-term spatial memory was preserved and that the reduced number of alternations mainly reflected lower locomotor/exploratory activity rather than a short-term spatial memory deficit (Fig. 2C).

MWM training phase showed a significant day × genotype interaction (*F*_1.8, 69.45_ = 7.193, *p =* 0.002), with *Negr1*^–/–^ mice requiring more time to reach the hidden platform in both sexes. (Fig. 2D). In the probe trials phase, the analysis showed a significant age × genotype interaction (*F*_1, 30_ = 6.575, *p =* 0.016) (Fig. 2D). Although no significant differences were observed at 12 weeks between genotypes (*p* = 0.494), by 18 weeks, a significant main effect of genotype emerged (*p* = 0.028), with *Negr1*^–/–^ mice spending significantly less time in the target quadrant compared to WT mice, indicating progressive impairment in spatial memory retention.

However, SPT revealed no significant effects of genotype, sex, or their interaction (*F*_1, 20_ = 0.907, *p =* 0.496), suggesting that anhedonia-like behavior was not prominent in this model (Supplementary Fig. S2E).

These results demonstrated that, in both sexes, the absence of *Negr1* impairs social interaction and long-term spatial learning and memory, and increases anxiety-like behavior.

### Sex-specific behavioral phenotypes of *Negr1*^–/–^ mice

We found that impairments of long-term spatial memory as well as social interaction accompanied by anxiety-like behaviors were common phenotypes in *Negr1*^–/–^ mice in both male and female mice.

**Fig. 3:**
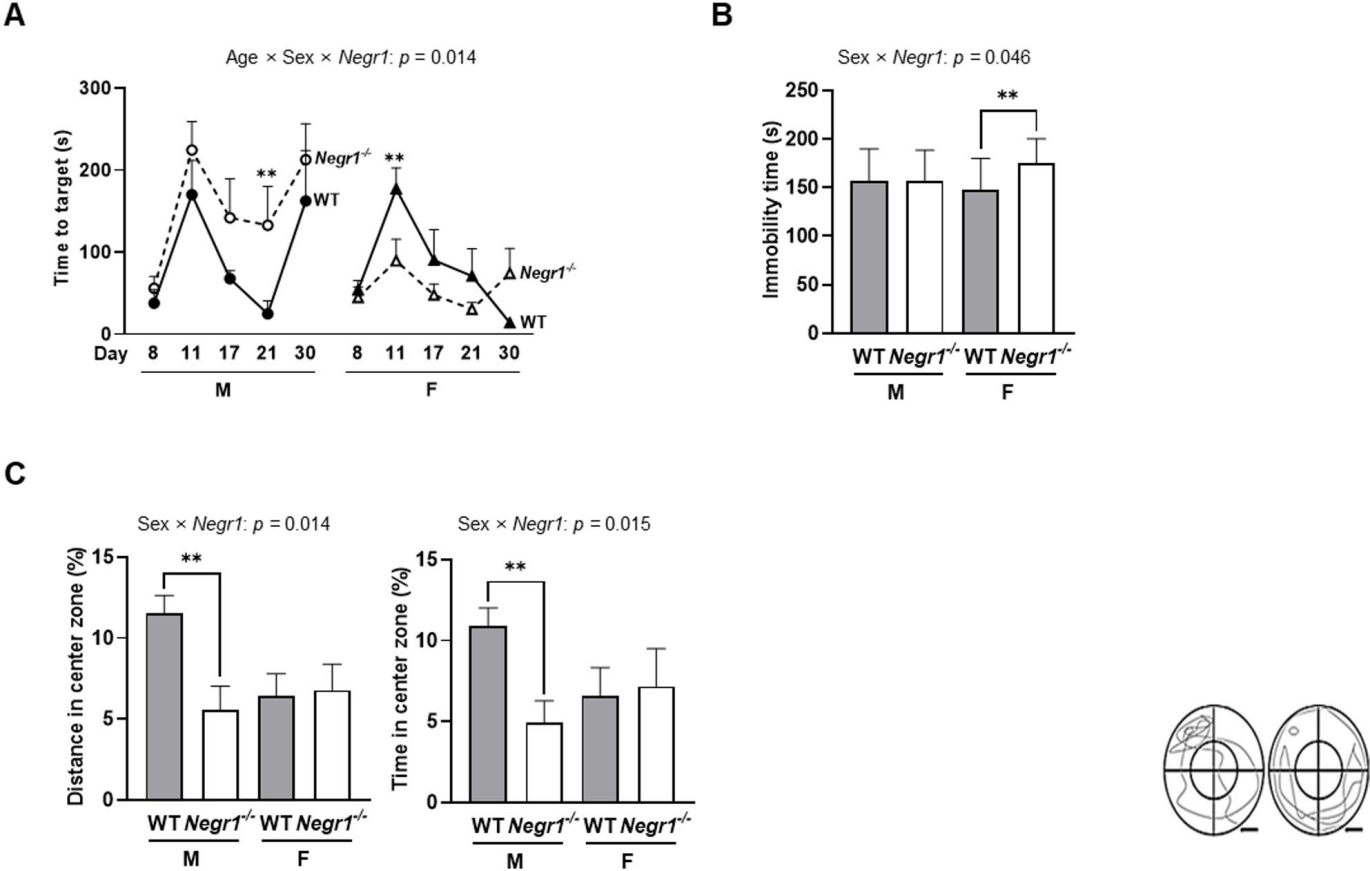
Female *Negr1*^*–/–*^ mice exhibited greater depression-like behavior and impaired fear learning, whereas male *Negr1*^*–/–*^ mice exhibited greater anxiety. **(A)** Assessment of latency time in the passive avoidance test (PAT). **(B)** Assessment of immobility time in the tail suspension test (TST). **(C)** Assessment of open arm distance (%) and time (%) relative to total distance and time, measured in the elevated plus maze (EPM). Data in this figure were obtained from Cohort 3, which underwent repeated behavioral assessments (8–30 weeks). Panel A presents longitudinal data to reflect the significant age x sex x genotype interaction observed. Panel B and C present time-collapsed summary data across the tested ages, as only a significant sex x genotype interaction was observed without a significant age interaction. All data are presented separated by sex to highlight the significant sex x genotype interactions. RM-ANOVA was used to assess group main effects and interactions, with Sidak’s post hoc comparisons. WT vs. *Negr1*^–/–^ comparisons: ** *p <* 0.01. Data are presented as mean ± SEM. M, males; F, females

However, in the PAT, RM-ANOVA analysis revealed a significant age × sex × genotype interaction (*F*_4, 112_ = 3.277, *p =* 0.014) (Fig. 3A). Male *Negr1*^–/–^ mice exhibited the pattern of longer latency times than male WT mice and were significantly longer at 21 weeks (*p <* 0.01), indicating enhanced fear-memory retention in the absence of *Negr1* in males. In contrast, female *Negr1*^–/–^ mice exhibited significantly shorter latency times compared to female WT mice at 11 weeks (*p <* 0.01), indicating impaired fear-memory retention in females. Interestingly, female *Negr1*^–/–^ mice showed most fearless behavior toward foot shock among other groups.

The TST is the most representative behavioral test for depression in vivo. Our study showed a significant sex × genotype interaction in TST (*F*_1, 24_ = 4.438, *p =* 0.046). Female *Negr1*^–/–^ mice showed significantly increased immobility time compared with female WT controls (*p =* 0.006), indicating depression-like behavior in female *Negr1*^–/–^ mice, while no such differences were observed in males (Fig. 3B).

In the EPM, significant sex × genotype interactions were observed for both open arm distance (*F*_1, 30_ = 6.868, *p =* 0.014) and time (*F*_1, 30_ = 6.591, *p =* 0.015). Male *Negr1*^–/–^ mice showed significantly reduced distance (*p =* 0.003) and time (*p =* 0.005) spent in open arms compared with male WT controls, indicating heightened anxiety-like behavior in male *Negr1*^–/–^ mice, while no significant differences were observed in females (Fig. 3C). Total distance traveled showed no significant genotype differences (*F*_1, 30_ = 3.934, *p* = 0.057), confirming that reduced open arms exploration was not due to locomotor impairments (Supplementary Fig.S3C).

These results demonstrate distinct sex-dependent responses where females exhibit greater vulnerability to depression-like behaviors and impaired fear learning, while males show more anxiety-like responses to height.

### Sex-specific differences in ER stress markers in *Negr1*^–/–^ mice

To investigate the molecular mechanisms underlying the observed sex-specific behavioral phenotypes, we examined ER stress markers in the brain, liver, and colon tissues.

**Fig. 4:**
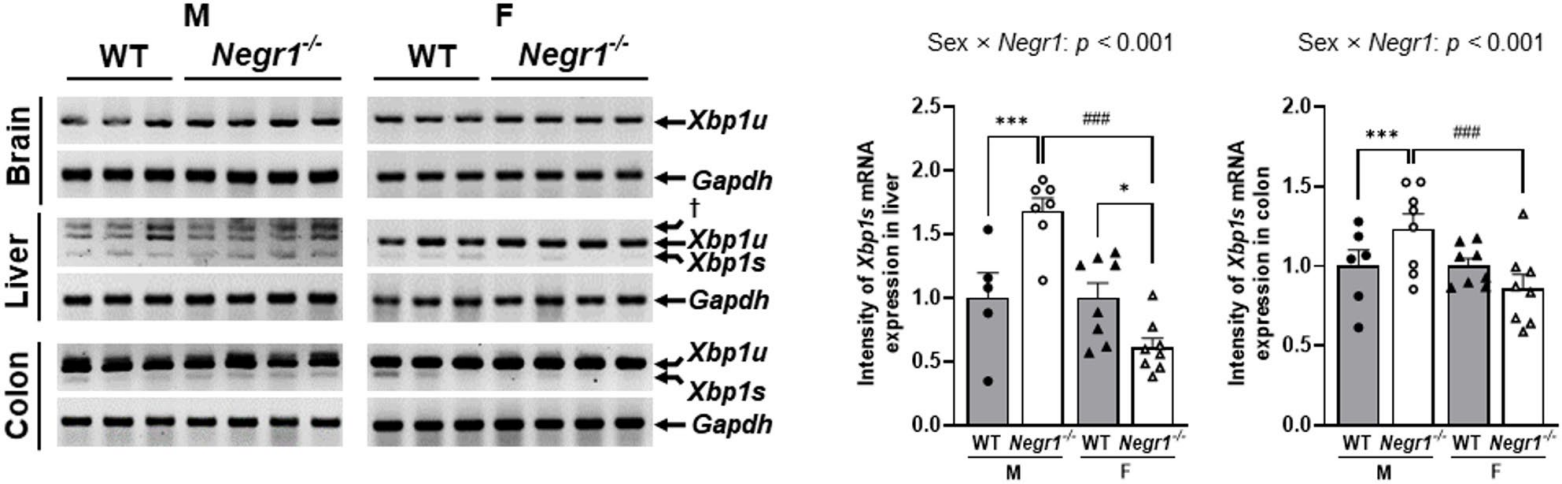
*Negr1*^–/–^ mice exhibit sex-specific regulation of ER stress. *XBP1s* mRNA expression in the brain, liver, and colon measured by RT-PCR. mRNA expression was quantified using ImageJ. Two-way ANOVA was used to assess group main effects and interactions, with Sidak’s post hoc comparisons. Male vs. female comparisons: ^###^ *p <* 0.001. WT vs. *Negr1*^–/–^ comparisons: *** *p <* 0.001. Data are presented as mean ± SEM. M, males; F, females; †: non-specific band

Analysis of *XBP1s* mRNA expression revealed significant sex × genotype interactions in the liver and colon tissues (liver: *F*_1, 26_ = 9.478, *p <* 0.001; colon: *F*_1, 26_ = 5.047, *p* = 0.025) but not in the brain (Fig. 4).

In both liver and colon, males *Negr1*^–/–^ mice exhibited significantly higher *Xbp1s* expression compared with female *Negr1*^–/–^ mice (liver: *p <* 0.001; colon: *p <* 0.01), and compared with male WT controls (liver: *p <* 0.001; colon: *p <* 0.01). Female *Negr1*^–/–^ mice showed significantly reduced expression compared with female WT controls in liver tissue (*p <* 0.05). This indicates opposite ER stress responses between the sexes, with males showing upregulated pro-survival pathways and females showing downregulated responses in peripheral tissues.

Analysis of other ER stress markers, including p-eIF2α protein levels (brain: *F*_1, 23_ = 0.045, *p* = 0.834; liver: *F*_1, 26_ = 2.906, *p* = 0.100; colon: *F*_1, 23_ = 0.009, *p* = 0.926) and *Chop* mRNA expression (brain: *F*_1, 24_ = 0.111, *p* = 0.742; liver: *F*_1, 24_ = 0.004, *p =* 0.953; colon: *F*_1, 26_ = 0.168, *p* = 0.685), revealed no significant sex × genotype interactions across all tissues examined (Supplementary Fig. S4).

### Sex-specific differences in apoptosis markers in *Negr1*^–/–^ mice

Given the differential ER stress responses between the sexes, we examined caspase-3 activation as a marker of apoptotic signaling. Western blot analysis revealed striking sex-specific patterns of caspase-3 expression and activation across different tissues.

**Fig. 5:**
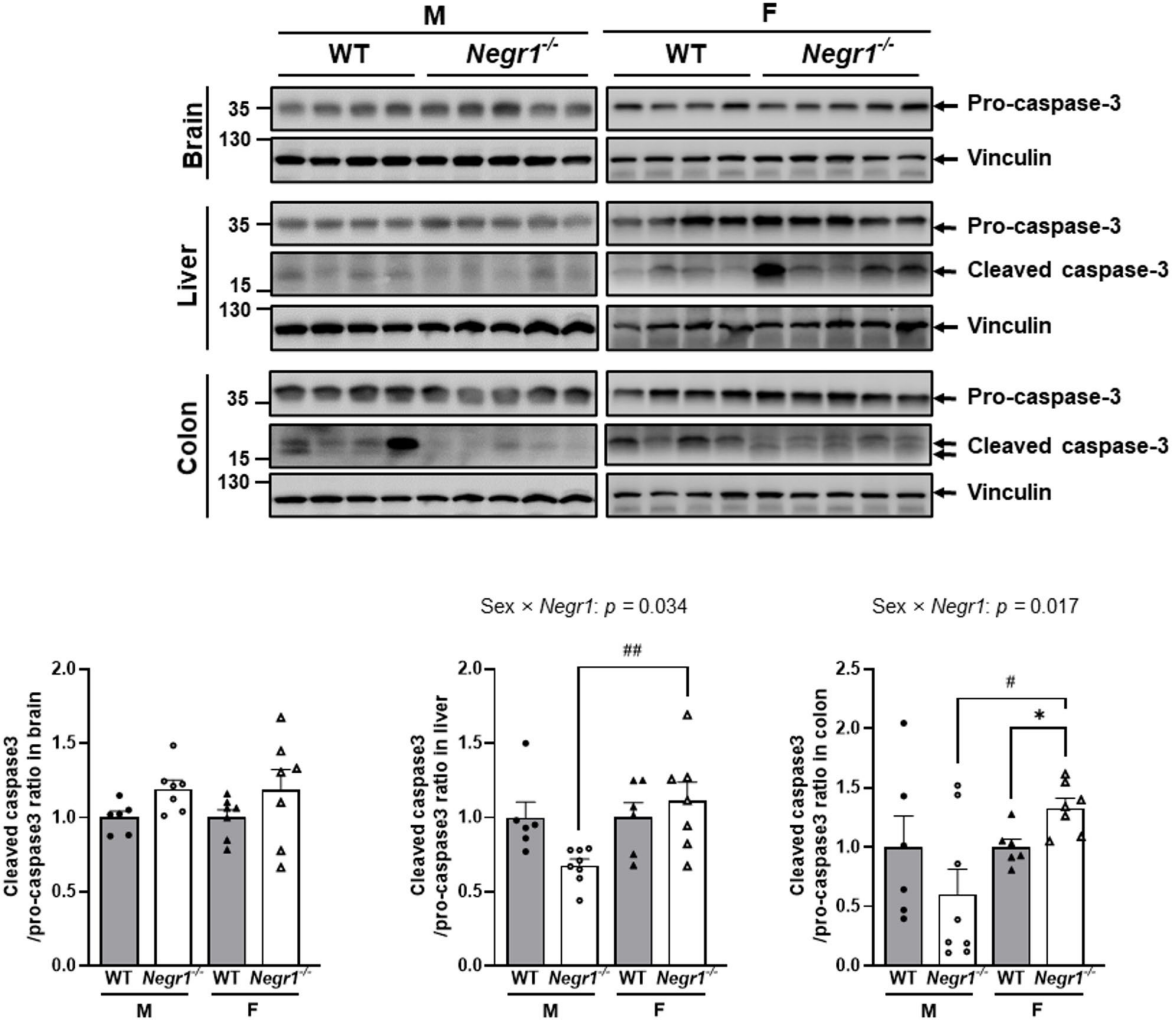
*Negr1*^–/–^ mice exhibit sex-specific regulation of apoptosis. Caspase-3 protein expression in the brain, liver, and colon measured by Western blot and quantification of caspase-3 protein levels in tissues. Normalization to loading controls for brain pro-caspase-3 (left) and cleaved/pro-caspase-3 ratios in liver (middle) and colon (right). Protein expression were quantified in ImageJ. Two-way ANOVA was used to assess group main effects and interactions, with Sidak’s post hoc comparisons. Male vs. female comparisons: ^#^ *p* < 0.05 and ^##^ *p* < 0.01. WT vs. *Negr1*^–/–^ comparisons : * *p* < 0.05 and *** *p* < 0.001. Data are presented as mean ± SEM. M, males; F, females

In the brain tissue, two-way ANOVA revealed no significant sex × genotype interactions for either pro-caspase-3 expression (*F*_1, 26_ = 2.824, *p* = 0.105), indicating that central apoptotic signaling was not significantly sex-dependently altered by *Negr1* absence (Fig. 5).

In both liver and colon, the analysis of the cleaved caspase-3 to pro-caspase-3 ratio revealed significant sex × genotype interactions (liver: *F*_1, 23_ = 5.054, *p =* 0.034; colon: *F*_1, 23_ = 6.647, *p* = 0.017), with female *Negr1*^–/–^ mice exhibiting significantly higher ratios compared with male *Negr1*^–/–^ mice (liver: *p <* 0.01; colon: *p <* 0.05), indicating a sex-dependent effect of *Negr1* deficiency. However, post-hoc comparisons demonstrated tissue-specific effects of the *Negr1* knockout. In colon tissue, Female *Negr1*^–/–^ mice showed significantly elevated ratios compared with female WT controls (*p* = 0.05). Conversely, no significant difference was observed between female *Negr1*^–/–^ mice and female WT controls in the liver. No significant differences were observed between male groups or between WT groups in either tissue (Fig. 5).

These molecular findings reveal that *Negr1* absence triggers fundamentally different cellular responses between the sexes in peripheral tissues. While males show upregulated pro-survival ER stress pathways with no significant changes in apoptotic signaling, females exhibit downregulated pro-survival responses coupled with enhanced apoptotic activation, suggesting increased cellular vulnerability that may contribute to their greater susceptibility to depression-like behaviors.

## Discussion

Depression is more prevalent among women than men worldwide, with over 10% of pregnant and postpartum women experiencing depression [30, 31]. This well-known sex difference can be attributed to various psychological, physiological, social, and cultural factors. In this study, we investigated the sex-specific mechanisms underlying depression using *Negr1*^–/–^ mice, which exhibit a depressive-like phenotype. Our findings demonstrate distinct patterns of behavioral and molecular changes between male and female *Negr1*^–/–^ mice, suggesting sex-specific pathophysiological mechanisms in depression.

While both sexes showed general behavioral retention in social interaction, long-term spatial learning and memory in the absence of *Negr1*, female *Negr1*^–/–^ mice displayed impaired fear learning and increased vulnerability to depression-related behaviors. In contrast, male *Negr1*^–/–^ mice showed heightened anxiety-like behavior, although locomotor activity was unaffected across all tests, confirming that these behavioral phenotypes reflect genuine alterations in emotional and cognitive processes rather than motor impairments. In this study, we observed partially different patterns of anxiety-like behavior between the OFT and EPM in *Negr1*^–/–^ mice. In the OFT, only a significant main effect of genotype was observed, indicating that *Negr1*^–/–^ mice exhibited a general tendency toward increased anxiety-like behavior, regardless of sex. In contrast, a sex × genotype interaction was observed in the EPM, with a more pronounced increase in anxiety-like behavior in male *Negr1*^–/–^ mice compared to other groups. Previous studies have shown that the OFT is more sensitive to acute, context-dependent anxiety responses to novel environments (state anxiety) [32], while the EPM serves as an indicator of risk avoidance and more stable anxiety tendencies (trait-like anxiety) [33]. Therefore, the partial discrepancy observed in the OFT and EPM could be interpreted as a result of *Negr1* deficiency affecting state and trait anxiety in different ways and to different degrees. In other words, while *Negr1* deficiency may overall increase acute anxiety responses to novel environments (state anxiety) in both males and females, more persistent risk avoidance and trait-like anxiety tendencies tend to be more pronounced in males.

These sex-specific differences highlight distinct neural and molecular adaptations in response to absence of *Negr1*. The stronger depression-like phenotypes in females and anxiety-like phenotypes in males point to divergent mechanisms that may involve sex hormones, neuroplasticity, and peripheral–central communication. These results underscore the importance of sex as a biological variable in studies of *Negr1* and related pathways, and may inform the development of sex-specific therapeutic approaches for depression and anxiety disorders.

The sex-specific behavioral phenotypes we observed may be explained, at least in part, by differential changes in neuroplasticity markers, particularly *Bdnf* expression. At 8 weeks, a significant sex difference was observed within the *Negr1*^–/–^ group, with males showing higher *Bdnf* expression than females. This early sex-specific difference in *Bdnf* expression among *Negr1*^–/–^ mice may reflect differential neuroplastic response between the sexes from an early developmental stage.

At 18 weeks in Cohort 2, male WT mice showed significantly higher *Bdnf* expression than male *Negr1*^–/–^ mice, resulting in lower *Bdnf* levels in male *Negr1*^–/–^ mice compared with female *Negr1*^–/–^ mice. A similar sex × genotype interaction was observed at 32 weeks in Cohort 3, where *Bdnf* mRNA was measured after repeated behavioral testing, with male WT mice again showing higher *Bdnf* expression than male *Negr1*^–/–^ mice, while genotype differences were not evident in females. Although experimental conditions differed between cohorts, the finding that male *Negr1*^–/–^ mice showed relatively lower *Bdnf* expression at both 18 and 32 weeks in both cohorts suggests that impairment of BDNF-dependent neuroplasticity may be related to a greater vulnerability to anxiety-related behaviors in males. BDNF is a key regulator of neuroplasticity, and its reduction in the hippocampus and prefrontal cortex has been consistently associated with depressive-like behaviors in rodent models and depression in humans [26, 27]. The progressive decline in *Bdnf* expression in male *Negr1*^–/–^ mice may contribute to the sustained anxiety-like behaviors observed across multiple time points.

In contrast, female *Negr1*^–/–^ mice showed no significant changes in *Bdnf* expression at any age, suggesting that alternative mechanisms, rather than sustained *Bdnf* dysregulation, likely underlie the depression-like behaviors observed in females. Recent studies have identified sex-specific alterations in depression-relevant circuits, including the ventral hippocampus–nucleus accumbens (vHPC–NAc) pathway [34] and lateral habenula–midbrain dopamine pathways encompassing projections to the ventral tegmental area (LHb–VTA) [35], that contribute to female vulnerability to depression. These circuit-level changes, potentially mediated by sex hormones, may play a more prominent role in female *Negr1*^–/–^ mice and warrant future investigation.

Chronic stress and anxiety can activate the hypothalamic-pituitary-adrenal (HPA) axis, leading to the release of stress hormones like CORT [36, 37]. In our *Negr1*^–/–^ mouse model, however, we found that serum CORT levels showed no significant genotype effects, suggesting the behavioral phenotypes are independent of HPA axis dysregulation caused by absence of *Negr1*. This finding aligns with previous studies indicating that the anxiety-like behaviors observed in *Negr1*^–/–^ mice were not caused by compromised HPA axis function [13]. We did observe a significant main effect of sex, with males showing lower serum CORT levels than females regardless of genotype. This pattern is consistent with previous reports that female rodents generally exhibit a more pronounced HPA axis response to stress than males [38]. These observations suggest that the behavioral phenotypes in our study likely stem from neurobiological mechanisms independent of HPA axis dysregulation, though the CORT measurements performed at 32 weeks of age, after repeated behavioral testing, may reflect cumulative stress effects rather than baseline HPA axis function. Therefore, the increase in CORT observed in Cohort 3 is likely due to the cumulative influence of repeated behavioral testing.

One of our most striking findings was the opposite regulation of cellular stress responses between male and female *Negr1*^–/–^ mice in peripheral tissues. The significantly upregulated *Xbp1s* mRNA in the liver and the colon of male *Negr1*^–/–^ mice, contrasted with downregulation in female *Negr1*^–/–^ mice, reveals fundamentally different cellular stress responses between the sexes. XBP1s is a critical transcription factor in the adaptive unfolded protein response pathway, promoting cell survival under ER stress conditions [39]. The upregulation observed in males suggests activation of pro-survival mechanisms, while downregulation in females suggests impaired adaptive responses that could render them more vulnerable to cellular damage over time. The absence of significant changes in other ER stress markers (p-eIF2α and *Chop*) in our study suggests that *Negr1* absence selectively affects specific branches of the ER stress response rather than inducing global ER stress. This selective activation of XBP1s-mediated pathways may have important functional implications, as recent research has shown that sustained overexpression of Xbp1s in forebrain excitatory neurons leads to recurrent spontaneous seizures and sudden death in mice [40], suggesting that neuronal *Xbp1s* is maintained at very low levels under normal conditions.

The sex-specific differences in apoptotic activation provide further insight into the divergent pathophysiological mechanisms. In peripheral tissues, female *Negr1*^–/–^ mice exhibited significantly higher cleaved caspase-3 to pro-caspase-3 ratios compared with male *Negr1*^–/–^ mice, indicating enhanced apoptotic activity specifically in females. In contrast, brain tissue showed no significant alterations in caspase-3 activation in either sex, suggesting that the sex-specific apoptotic differences are primarily peripheral phenomena. The enhanced apoptotic activity in peripheral tissues of female *Negr1*^–/–^ mice may contribute to their depression-like behavioral phenotype through several potential mechanisms. First, increased peripheral apoptosis could lead to the release of damage-associated molecular patterns (DAMPs) that trigger systemic inflammation [41]. These inflammatory signals can influence central nervous system function through multiple pathways, including cytokine signaling across the blood-brain barrier and vagus nerve activation, both of which have been implicated in depression pathogenesis [42]. Second, apoptosis plays a crucial role in maintaining tissue homeostasis, and accelerated apoptosis has been associated with stress-induced depression in clinical and animal studies [43, 44]. The combination of enhanced peripheral apoptosis and impaired pro-survival responses in female *Negr1*^–/–^ mice may therefore create a systemic environment that promotes depression-like behaviors, potentially explaining the increased immobility observed in the TST and impaired fear learning in the PAT.

Our investigation of peripheral tissues revealed important sex-specific differences that may contribute to the observed behavioral phenotypes through the gut–brain axis. The increased intestinal permeability in *Negr1*^–/–^ mice, regardless of sex, indicates compromised gut barrier function that could allow bacterial products and inflammatory mediators to enter the circulation [45]. This increase in permeability may influence behavior through neuroinflammatory pathways [46]. However, because the degree of permeability is similar in males and females, barrier dysfunction alone cannot explain the sex-specific behavioral differences, suggesting the involvement of additional sex-specific factors. The sex-specific differences in peripheral ER stress and apoptotic responses described above likely interact with intestinal barrier dysfunction to create distinct patterns of gut–brain axis communication in males and females. The combination of increased intestinal permeability and upregulated pro-survival ER stress pathways in males may represent an attempt to maintain peripheral tissue homeostasis. In contrast, females showed increases in both apoptosis and intestinal permeability. Epithelial damage can increase intestinal permeability even in the absence of detectable changes in tight junction protein expression [47, 48]. Therefore, in females, although the expression of tight junction related mRNAs (*Muc2*,* occludin*,* claudin-2*,* Zo-1*) was unchanged (Supplementary Fig. S5), the observed increases in intestinal permeability and apoptosis are likely to reflect epithelial injury, which may exacerbate peripheral tissue damage and enhance inflammatory signaling to the brain.

While we did not directly measure estrogen levels or estrogen receptor expression in our study, our findings are consistent with the known anti-inflammatory and neuroprotective effects of estrogen. Estrogen receptor β (ERβ) agonists have been shown to attenuate the IRE1/XBP1 pathway, thereby mitigating ER stress-induced deficits in social behavior and brain connectivity in mice [49]. The sex-specific differences in *Xbp1s* regulation we observed may reflect differential hormonal modulation of cellular stress responses, with estrogen potentially providing some protective effects in females under certain conditions. However, the eventual development of enhanced apoptosis and more severe behavioral deficits in female *Negr1*^–/–^ mice suggest that these protective mechanisms are insufficient to fully compensate for *Negr1* absence. Meanwhile, recent studies have reported that male *Negr1*^–/–^ mice show reduced testosterone, thereby exacerbating sexual dysfunction and anxiety- and depressive-like behaviors, and that testosterone supplementation partially reverses these behavioral deficits [50]. This evidence supports the possibility of a causal link between sex hormones and behavioral phenotypes.

This model has important implications for the treatment of anxiety and depression, suggesting that interventions targeting peripheral ER stress responses and apoptosis might be particularly beneficial for females, while approaches addressing central neuroplasticity and anxiety circuits might be more effective for males. Moreover, the sex-specific nature of these mechanisms highlights the critical importance of considering sex as a biological variable in both preclinical research and the development of clinical treatments.

Several limitations of our study should be acknowledged. First, we did not control for the estrous cycle in female mice, which could potentially influence behavioral outcomes. However, recent studies indicate that the estrous cycle may not have a decisive impact on behavioral outcomes in depression-related tests [51, 52]. Nevertheless, future studies incorporating estrous cycle monitoring would provide additional insights into the potential modulatory effects of cyclical hormonal changes on depression-related behaviors. Second, our mechanistic analyses were performed at 32 weeks, when behavioral differences between the sexes were established. Longitudinal molecular analyses at multiple time points, particularly during the early stages when sex-specific behavioral differences first emerge, would provide a more comprehensive understanding of the temporal dynamics of these molecular changes and help establish causal relationships with behavioral phenotypes. Third, a more comprehensive analysis of additional tissues, signaling cascades, and the gut microbiome composition would provide a more complete picture of the systemic effects of *Negr1* absence. Future studies should investigate whether *Negr1* is expressed in intestinal tissues and how its absence affects gut homeostasis in a sex-specific manner. Finally, our study did not include pharmacological interventions targeting the identified pathways. Future studies employing modulators of ER stress responses, apoptotic pathways, or estrogen signaling could provide causal evidence for the role of these mechanisms in the sex-specific behavioral phenotypes observed in *Negr1*^–/–^ mice, and potentially identify new therapeutic targets for anxiety and depression.

## Conclusion

Our study demonstrates significant sex differences in the behavioral and molecular phenotypes of *Negr1*^–/–^ mice. While both sexes showed general behavioral alterations in social interaction, females exhibited greater vulnerability to depression-like behaviors and impaired fear learning, whereas males showed heightened anxiety-like responses. These behavioral differences are associated with opposite peripheral ER stress responses and differential apoptotic signaling between the sexes, suggesting fundamentally different cellular mechanisms. Our findings highlight the importance of considering sex as a biological variable in depression research and suggest that *Negr1* plays a crucial role in the sex-specific pathophysiology of psychiatric disorders through complex mechanisms spanning central and peripheral systems. Understanding these sex-specific mechanisms may lead to more personalized approaches to diagnosis and treatment of anxiety and depression disorders.

## Supplementary Information

Below is the link to the electronic supplementary material.

Supplementary Material 1.

## Data Availability

All data generated or analyzed during this study are included in this published article and its supplementary information file.
