# Supplementary material for: Sex-specific difference on anxiety- and depressive-like behavior in neuronal growth regulator 1-knockout mice
Source: Biol Sex Differ. 2026 Jan 26;17:31. doi: 10.1186/s13293-025-00816-2 (PMC12918530; doi:10.1186/s13293-025-00816-2)
Supplement: Supplementary file 1 — Supplementary Material 1. [file 13293_2025_816_MOESM1_ESM.docx]

**Sex-specific difference on anxiety- and depressive-like behavior in Neuronal growth regulator 1-knockout mice**

So Rok Lee^1^, Eunji Yoon^2^, Sooyeon Baek^1^, Jin Gyeom Kim^1,5^, Jong-Oh Kim^3^, Su-In Yoon^1, 2^, Soojin Lee^4^, Jin Ah Cho^1, 5, *^

**Supplementary Table. S1 Target genes and primer sequences for mouse RT-qPCR**

| **Gene** | **Primer** | **Sequence (5′→3′)** |
| --- | --- | --- |
| *Bdnf* | forward | TGA GTC TCC AGG ACA GCA AA |
|  | reverse | GCC TTC ATG CAA CCG AAG TA |
| *Muc2* | forward | GCC TGT TTG ATA GCT GCT ATG TGC C |
|  | reverse | GTT CCG CCA GTC AAT GCA GAC AC |
| *Occludin* | forward | ACC CGA AGA AAG ATG CAT CG |
|  | reverse | CAT AGT CAG ATG GGG GTG GA |
| *Claudin-2* | forward | CCT TCG GGA CTT CTA CTC GC |
|  | reverse | TCA CAC ATA CCC AGT CAG GC |
| *Zo-1* | forward | AAG AAA AAA GAA TGC ACA GAG TTG TT |
|  | reverse | GAA ATC GTG CTG ATG TGC CA |
| *Gapdh* | forward | ACA ACT TTG GCA TTG TGG AA |
|  | reverse | GAT GCA GGG ATG ATG TTC TG |

**Supplementary Table. S2 Target genes and primer sequences for mouse RT-PCR**

| **Gene** | **Primer** | **Sequence (5′→3′)** | **Base Pair (bp)** |
| --- | --- | --- | --- |
| *Xbp1* | forward | GAG TCC GCA GCA GGT G | *Xbp1u*:171 |

|  | reverse | GTG TCA GAG TCC ATG GGA | *Xbp1s*:145 |
| --- | --- | --- | --- |
| *Chop* | forward | CAC ATC CCA AAG CCC TCG CTC TC | 286 |
|  | reverse | TGC TTG GTG CAG GCT GAC CAT |  |
| *Gapdh* | forward | T CAC TGC CAC CCA GAA GAC TG | 147 |
|  | reverse | ATG CCA GTG AGC TTC CCG TTC AG |  |


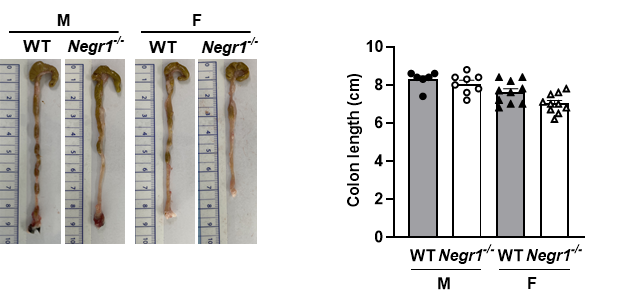


**Supplementary Figure. S1** *Negr1^−/−^* mice have colon lengths comparable to those of WT mice. Representative colon length image (left) and colon lengths for all samples (right). Two-way ANOVA was used to assess group main effects and interactions. Data are presented as mean ± SEM. M, males; F, females.


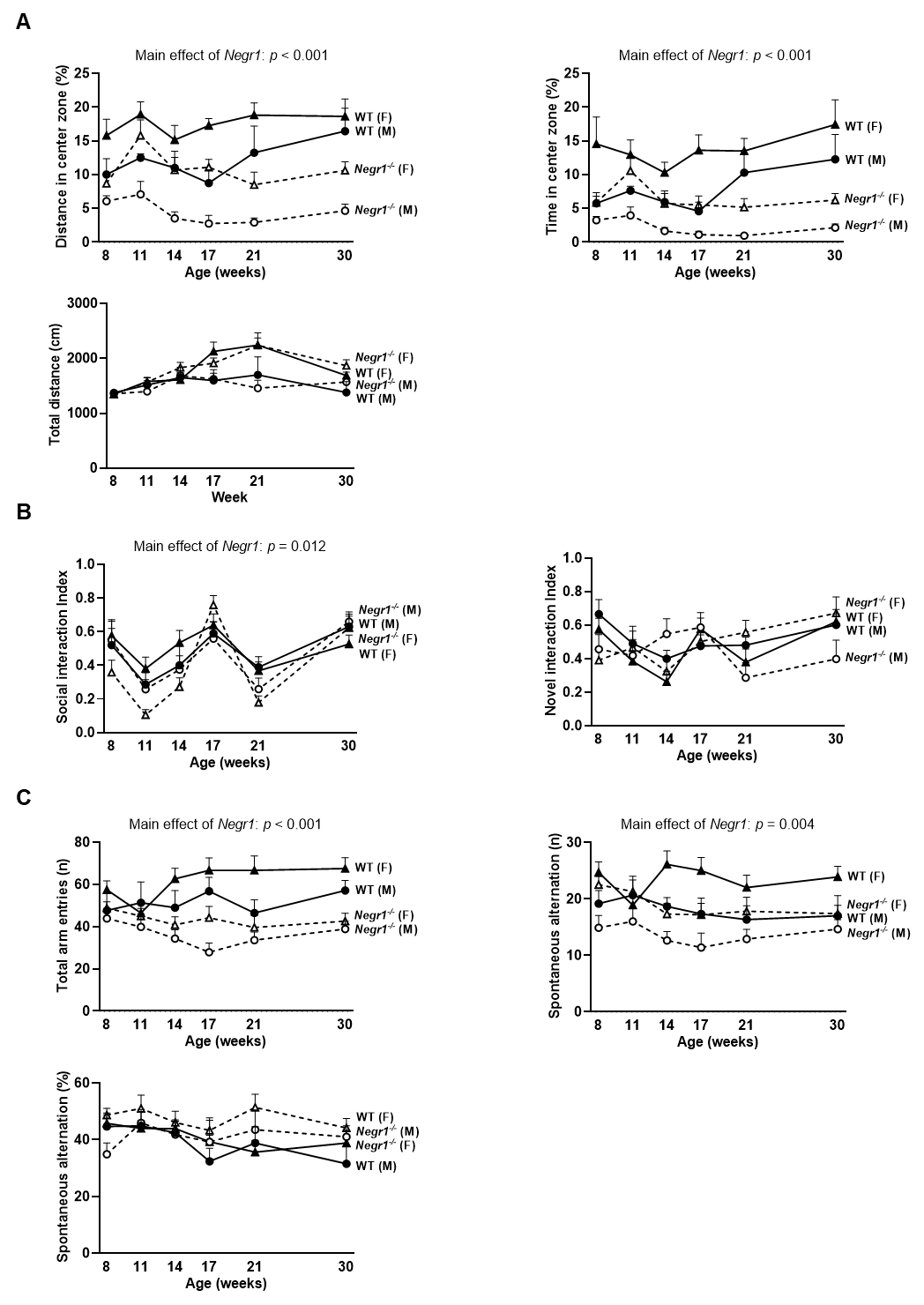


**
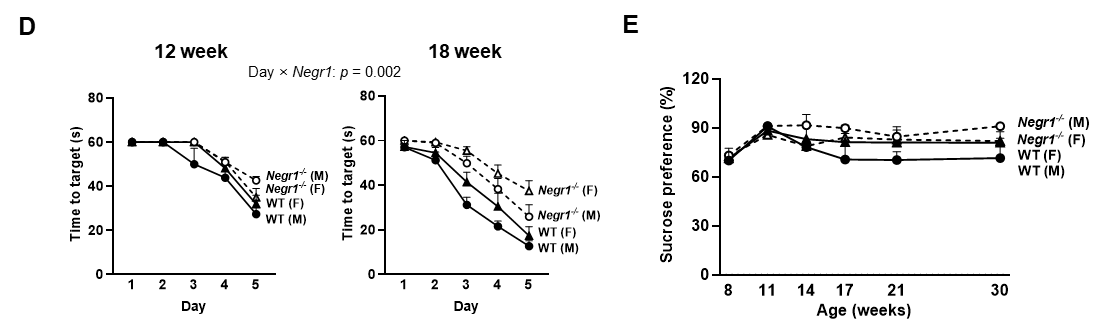
Supplementary Figure. S2** *Negr1^−/−^* mice exhibit behavioral phenotypes common to both sexes. Behavioral tests (OFT, 3-SIT, Y-maze and SPT) at weeks 8, 11, 14, 17, 21, and 30 across all groups. **A.** Evaluation of anxiety-like behavior using the OFT. **B.** Evaluation of social interaction and social novelty using the 3-SIT. **C.** Evaluation of short-term spatial memory using the Y-maze. **D.** Evaluation of spatial learning using the MWM at 12 and 18 weeks. **E.** Evaluation of anhedonia using the SPT. RM-ANOVA was used to assess group main effects and interactions, with Sidak’s post hoc comparisons. Data are presented as mean ± SEM. M, males; F, females.


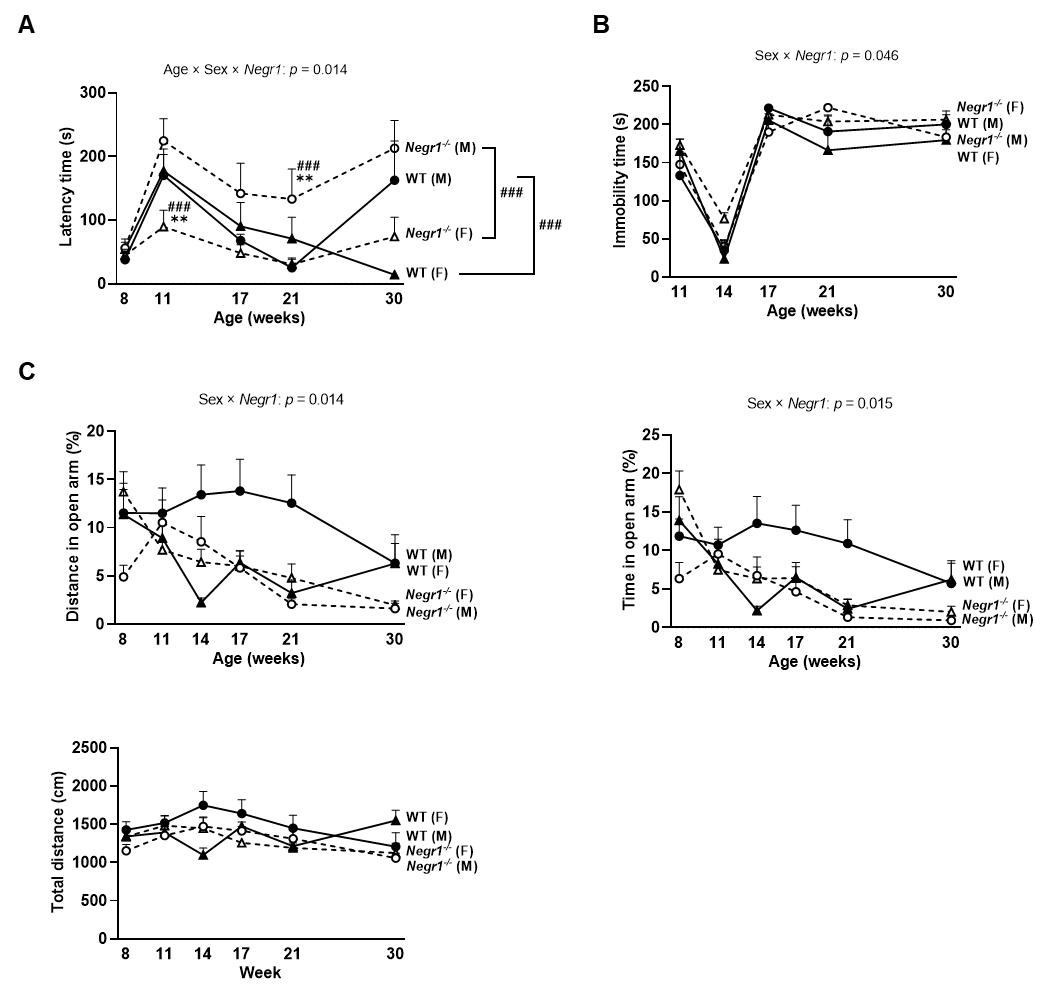


**Supplementary Figure. S3** *Negr1^−/−^* mice exhibit sex-specific behavioral phenotypes. **A.** Evaluation of fear learning using the PAT at 8, 11, 17, 21, and 30 weeks. **B.** Evaluation of depressive-like using the TST at 11, 14, 17, 21, and 30 weeks. **C.** Evaluation of anxiety-like using the EPM at 8, 11, 14, 17, 21, and 30 weeks. Male vs. female comparisons: ^###^ *p <* 0.001. WT vs. *Negr1*^–/–^ comparisons: ** *p <* 0.01. Data are presented as mean ± SEM. M, males; F, females.


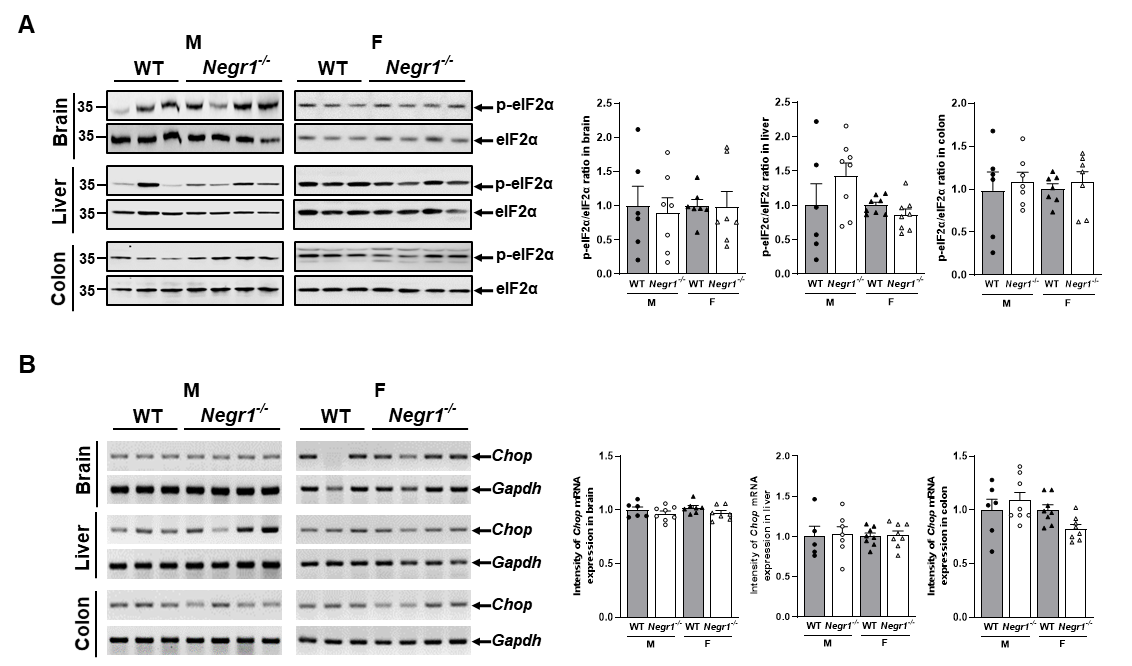


**Supplementary Figure. S4** *Negr1^−/−^* mice exhibit no sex-specific changes in eIF2α–CHOP related ER stress responses. **A.** p-eIF2α protein expression in the brain, liver, and colon measured by Western blot. Normalization to p-eIF2α/eIF2α ratios in brain, liver and colon. **B.** *Chop* mRNA expression in the brain, liver, and colon measured by RT-PCR. Protein and mRNA expression were quantified using ImageJ. Two-way ANOVA was used to assess group main effects and interactions. Data are presented as mean ± SEM. M, males; F, females.


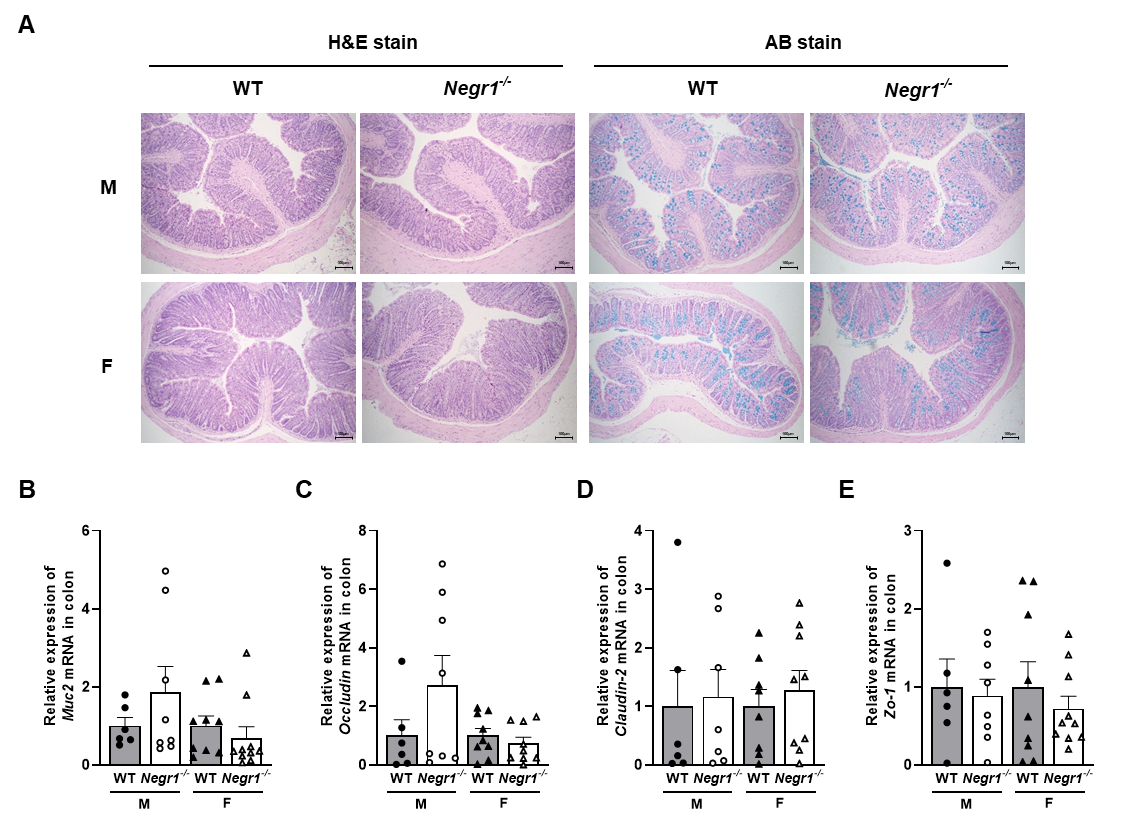


**Supplementary Figure. S5** *Negr1^−/−^* mice exhibit no sex-specific differences in colon structure or tight-junction expression. **A.** Representative H&E and AB staining of the colon. Scale bar =100 μm. **B-E.** *Muc2*, *Occludin*, *Claudin-2, Zo-1* mRNA expression in colon measured by RT-qPCR. Two-way ANOVA was used to assess group main effects and interactions. Data are presented as mean ± SEM. M, males; F, females.
